# US Residents’ Recognition of Proper Use of Firearm Cable Locks

**DOI:** 10.1001/jamanetworkopen.2024.15064

**Published:** 2024-06-05

**Authors:** Shelby L. Bandel, Jayna Moceri-Brooks, Allison E. Bond, Daniel Semenza, Michael D. Anestis

**Affiliations:** 1 New Jersey Gun Violence Research Center, Rutgers School of Public Health, West Piscataway, New Jersey; 2 Department of Psychology, Rutgers, The State University of New Jersey, West Piscataway, New Jersey; 3 Department of Sociology, Anthropology, and Criminal Justice, Rutgers University–Camden, Camden, New Jersey

## Abstract

This survey study examines the ability of firearm owners and nonowners to determine correct and incorrect cable lock use across different types of firearms.

## Introduction

Firearm-related injuries and fatalities are a public health concern.^1^ Secure firearm storage protects against these outcomes,^2^ yet many owners store firearms unsecured.^3^ Although cable locks are not preferred by firearm owners,^4,5^ their cost and availability render them optimal for broad distribution. However, cable locks must be installed correctly to be effective. This survey study examined firearm owners’ and nonowners’ ability to determine correct and incorrect cable lock use across different types of firearms.

## Methods

Study procedures were approved by the Rutgers Biomedical and Health Sciences Institutional Review Board; electronic consent was obtained from all participants. We followed the AAPOR reporting guideline for survey studies.

Eligible participants were older than 18 years, spoke English, and lived in Colorado, Minnesota, Mississippi, New Jersey, or Texas. Data were collected via an online survey between April 29 and May 15, 2022, using probability-based sampling from KnowledgePanel (Ipsos). Demographic data were collected via self-report to help characterize the data and to assist in data weighting procedures. Data were weighted for geodemographic distribution based on the 2019 American Community Survey (eMethods in Supplement 1).

Participants were shown photographs of a pistol, shotgun, rifle, and revolver in which cable locks were properly or improperly installed (Figure). Firearm ownership was assessed with the question “Do you currently own a firearm?” Descriptive statistical analysis was conducted using SPSS, version 29 (IBM Corp).

**Figure.**
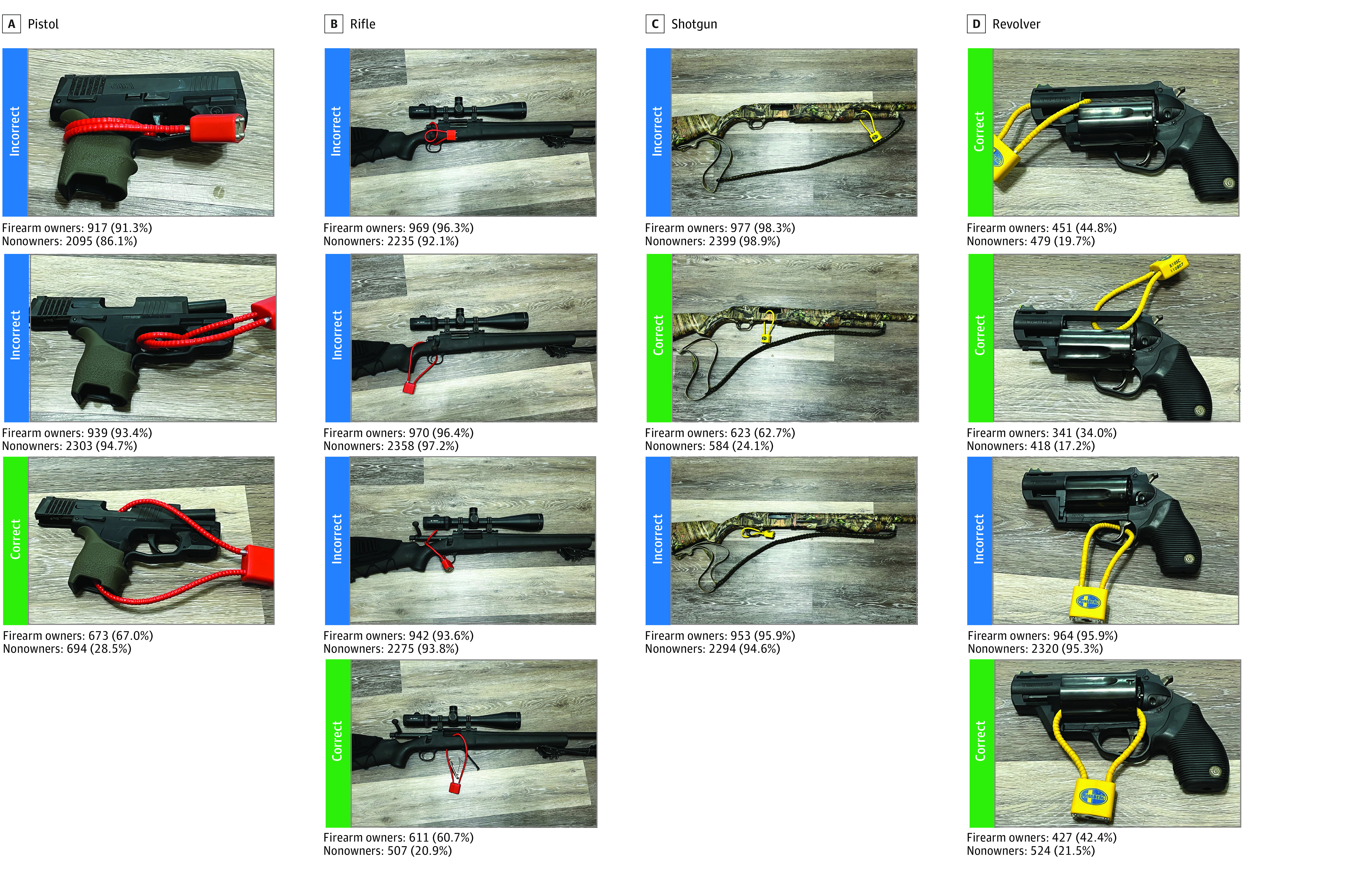
Photographs Shown to Participants With Correct and Incorrect Identification Includes 3462 respondents, consisting of 1016 firearm owners and 2446 nonowners. Percentages are weighted for geodemographic distribution based on the 2019 American Community Survey.

## Results

The study cohort included 3462 US adults with a mean (SD) age of 47.5 (17.0) years; 1684 (48.3%) were men, 1816 (51.7%) were women, and 1016 (29.3%) owned firearms (Table). In terms of race and ethnicity, 411 (11.7%) were Black, 912 (26.0%) were Hispanic, 1932 (55.0%) were White, 51 (1.5%) were multiracial, and 204 (5.8%) were of other race or ethnicity. (Table). Our results indicated gaps in owners’ (67.0%) and nonowners’ (28.5%) ability to determine correct lock use. Smaller knowledge gaps between groups were found when identifying incorrect lock use (93.4% and 94.7%, respectively). Individuals similarly identified correct and incorrect lock use on the rifle, handgun, and shotgun. However, gaps in knowledge about cable lock use on the revolver were greater than other types of firearms (Figure).

**Table.** Demographic Characteristics of the Study Participants

Characteristic	Data^a^
Age, y	
Mean (SD)	47.51 (17.03)
Range	18-94
Sex	
Male	1684 (48.3)
Female	1816 (51.7)
Race and ethnicity	
Black	411 (11.7)
Hispanic	912 (26.0)
White	1932 (55.0)
Multiracial	51 (1.5)
Other^b^	204 (5.8)
Firearm owner	
Yes	1016 (29.3)
No	2446 (70.7)

^a^ Unless otherwise indicated, data are expressed as No. (weighted %).

^b^ Individuals who self-identified as other race or ethnicity were presented with a text box to specify their race. Common responses included mixed, Mexican, and Hispanic.

## Discussion

This survey study examined individuals’ ability to accurately identify correct and incorrect cable lock installation across 4 types of firearms. The finding that individuals more accurately identified incorrect and correct lock use suggests that knowledge gaps may prompt incorrect lock use or avoidance of locks. Future efforts to distribute cable locks must include education on proper use.

Both firearm owners and nonowners displayed notable gaps in their knowledge of correct cable lock installation. Education about cable lock use at the point of firearm purchase may increase this knowledge among firearm owners. Future efforts to increase this knowledge regardless of firearm ownership status is also necessary. This is particularly salient, as previous research suggests that many individuals who have purchased firearms recently were first-time buyers.^6^

Difficulty determining proper cable lock use on the revolver might be related to cable locks being used differently on revolvers relative to other firearms. On revolvers, cable locks can be attached numerous ways, including looped through the barrel of the firearm, through one of the chambers on the cylinder, or around a part of the firearm (eg, the top strap) that would prevent the cylinder from connecting to the frame.

Notably, these results do not speak to an individual’s ability to install cable locks properly, only their ability to determine whether a lock is installed correctly. Future research examining people’s ability to install cable locks is necessary.

This survey study is the first, to our knowledge, to provide information on US residents’ ability to determine correct cable lock use. Our findings suggest that there are notable gaps in all individuals’ knowledge about how to use cable locks. Further efforts to provide education about proper use of cable locks on a variety of firearms is necessary to optimize use of this storage method.

## Notes

Supplement 1. **eMethods.** Design and Sample Weighting

Supplement 2.
Data Sharing Statement
