# Supplementary material for: US Residents’ Recognition of Proper Use of Firearm Cable Locks
Source: JAMA Netw Open. 2024 Jun 5;7(6):e2415064. doi: 10.1001/jamanetworkopen.2024.15064 (PMC11154157; doi:10.1001/jamanetworkopen.2024.15064)
Supplement: Supplement 1. — eMethods. Design and Sample Weighting [file jamanetwopen-e2415064-s001.pdf]

Supplementary Online Content

Bandel SL, Moceri-Brooks J, Bond AE, Semenza D, Anestis MD. US residents' recognition of proper use of firearm cable locks. *JAMA Netw Open*. 2024;7(6):e2415064. doi:10.1001/jamanetworkopen.2024.15064

eMethods. Design and Sample Weighting

This supplementary material has been provided by the authors to give readers additional information about their work.

© 2024 Bandel SL et al. *JAMA Network Open*.

eMethods. Design and Sample Weighting

The present study used data from a large online survey seeking to understand firearm ownership and related firearm behaviors within five states across the US (N = 3,510). Participants resided in New Jersey (n = 540), Mississippi (n = 178), Minnesota (n = 673), Texas (n = 1,704), and Colorado (n = 415). Participants were eligible if they resided in one of the five states, spoke English, and were over 18 years old. The five states were chosen because they represent diverse geographical regions, a range of political climates, and varying rates of firearm ownership. Participants were recruited by KnowledgePanel (KP) via Ipsos from April 29 and May 15, 2022. KP is an online panel of participants and is the largest and oldest probability-based online panel in the US. Additional information regarding KP can be found at: https://www.ipsos.com/en-us/solutions/public-affairs/knowledgepanel. The completion rate for the present study was 58%.

In addition to completing several other measures as part of the larger overall study, participants were shown 14 photos of firearms (three pistol photos, three shotgun photos, four rifle photos, and four revolver photos). For each type of firearm, at least one of the photos displayed the cable lock installed properly. For the pistol, shotgun, and rifle, correct cable lock installation meant that lock was looped through the magazine well and out the ejection port. Given that a revolver's loading mechanism differs from the other types of firearms, correct cable lock installation was distinct for this firearm relative to the pistol, shotgun, and rifle. Specifically, for a revolver, correct cable lock installation could mean the lock is looped through the barrel of the firearm, around the top strap, or through a chamber on the cylinder. For all firearms, additional photos that demonstrated the lock installed improperly (e.g., looped around the trigger guard of the firearm) were also displayed.

© 2024 Bandel SL et al. *JAMA Network Open*.

Photos were designed by the research team in consultation with a firearm owning military veteran who served as a subject matter expert. In addition to the normal weapons training, this expert also went through several specialized weapons training programs while serving in the Army. Similar backgrounds were chosen to make the photos as consistent as possible. The photos were designed, posed, and taken solely for this research project and the corresponding author owns all rights to these photos.

Participants were instructed to "select all photos where the cable lock has been properly placed on the firearm (i.e. would prevent the firearm from operating)." Therefore, participants selected photos where they believed the cable lock was properly installed and did not select photos where they believed the image represented an improperly installed cable lock.

Sample weighting.

The data were weighted for geodemographic distribution (race/ethnicity, gender by age, race/ethnicity by state, gender by state, education by state, and household income by state) in the five states based on the benchmarks from the 2019 American Community Survey. Weights were derived both relative to the full sample and to participants' state of residence. This study utilized the total sample weight for all analyses and demographic characteristics are presented in Table 1. Demographic characteristics for the sample were provided by Ipsos and the remaining variables (e.g., firearm ownership) were developed by the study team.

© 2024 Bandel SL et al. *JAMA Network Open*.
