# Supplementary material for: US Residents’ Recognition of Proper Use of Firearm Cable Locks
Source: JAMA Netw Open. 2024 Jun 5;7(6):e2415064. doi: 10.1001/jamanetworkopen.2024.15064 (PMC11154157; doi:10.1001/jamanetworkopen.2024.15064)
Supplement: Supplement 2. — Data Sharing Statement [file jamanetwopen-e2415064-s002.pdf]

Data Sharing Statement

Bandel. US Residents' Recognition of Proper Use of Firearm Cable Locks. *JAMA Netw Open*. Published June 05, 2024. doi:10.1001/jamanetworkopen.2024.15064

Data
Data available: No

Additional Information
Explanation for why data not available: The datasets used and/or analyzed during the current study are available from the corresponding author or mda141@sph.rutgers.edu on reasonable request.
